# Accuracy of a Three-Dimensional (3D)-Printed Patient-Specific (PS) Femoral Osteotomy Guide: A Computed Tomography (CT) Study

**DOI:** 10.3390/bioengineering9110667

**Published:** 2022-11-08

**Authors:** Maria Moralidou, Anna Di Laura, Johann Henckel, Harry Hothi, Alister James Hart

**Affiliations:** 1 Institute of Orthopaedics and Musculoskeletal Science, University College London, London HA7 4LP, UK; 2 Royal National Orthopaedic Hospital NHS Trust, Brockley Hill, Stanmore HA7 4LP, UK; 3 Department of Mechanical Engineering, University College London, London WC1E 7JE, UK

**Keywords:** 3D-printed patient-specific guides, total hip arthroplasty, femoral neck osteotomy

## Abstract

Femoral neck osteotomy creates a critical anatomical landmark for surgeons performing primary Total Hip Arthroplasty (THA); it affects the final height and position of the femoral component. Patient Specific Instrumentation (PSI) has been developed to guide the osteotomy. We aimed to assess the accuracy of a patient-specific (PS) femoral osteotomy guide in primary THA using three-dimensional (3D) computed tomography (CT) analysis. We included pre- and post-operative CT data of 103 THAs. All patients underwent 3D planning to define the optimal femoral neck osteotomy level. Our primary objective was to quantify the discrepancy between the achieved and planned osteotomy level; our secondary objective was to evaluate the clinical outcome. The median (Interquartile Range—IQR) discrepancy between the achieved and planned osteotomy level was 0.3 mm (−1 mm to 2 mm). We found a strong positive correlation between the planned and achieved osteotomy level (R^2^ = 0.9, *p* < 0.001). A satisfactory clinical outcome was recorded. Our findings suggest that surgeons can use 3D-printed PS guides to achieve a femoral neck osteotomy with a high level of accuracy to the plan.

## 1. Introduction

Optimal implant placement aims to restore the physiological hip function in primary Total Hip Arthroplasty (THA), but this is not always achieved [1,2,3]. There is considerable variability in the placement of the femoral stem; the version of an uncemented stem has been reported to range between −23° and 72° [4,5,6,7], whilst the vertical Femoral Offset (FO) discrepancy exceeding the clinically accepted limits of ±5 mm has been reported in 14–42% of THA patients [2]. In addition, a Leg Length Discrepancy (LLD) of more than 1 cm has been reported in 9–21% of patients [8,9], with up to 43% of patients perceiving the discrepancy [10]. 

Suboptimal placement of the femoral stem within the femur is associated with untoward clinical events, such as dislocation, altered gait kinematics, implant instability and inferior functional outcome [10,11,12,13,14,15,16]. During surgery, the femoral neck osteotomy constitutes the entry point of the stem and affects its final position and orientation, varus/valgus alignment and version [4,17].

Surgeons use either free-hand osteotomy [18], robotic arm [19] or Three-Dimensional (3D)-printed Patient-Specific Instruments (PSI) [20,21] to execute the osteotomy. Free-hand femoral neck osteotomy is typically performed by surgeons using their experience and finger measurements, potentially inducing uncertainty about the reproducibility of the planned femoral neck osteotomy level [18]. PSI has been developed to guide the execution of the surgical virtual plan.

A great number of studies have assessed the accuracy of PSI for knee osteotomy [22,23,24,25,26,27,28,29,30,31,32,33,34,35,36], while very few studies have assessed the accuracy of PS femoral osteotomy guides [21]. A potential explanation may be that there are more commercially available PSI cutting jigs for knee arthroplasty than for hip arthroplasty. These studies mainly used Two-Dimensional (2D)-based methods, such as conventional radiographs. However, conventional radiography may not offer a reliable illustration of the patient’s anatomy, when compared to the 3D nature of computed tomography (CT) [37,38]. 

We aimed to assess the accuracy of a commercially available PS femoral osteotomy guide in primary THA using 3D-CT analysis. Our primary objective was to evaluate the discrepancy between the achieved and planned osteotomy level. Our secondary objective was to evaluate the clinical outcome.

## 2. Materials and Methods

### 2.1. Study Design

This was a case cohort study involving a total of 107 patients (118 hips) undergoing primary THA due to osteoarthritis (OA) between February 2017 and December 2021. A PS femoral osteotomy guide was designed and 3D-printed for each case, designed according to the pre-operative CT data. The stereolithography (STL) file of the surgical plan (planned femoral neck osteotomy) was not available in 3 cases to allow comparison with the achieved osteotomy. Additionally, the PS guide was not used intra-operatively in 12 patients because the sterilization process was judged insufficient. This resulted in 103 cases where the PS guide was used. Pre- and post-operative CT scans of the patients were used to perform 3D analysis of osteotomy level. Planned versus achieved osteotomy levels were measured in terms of their relative vertical difference. 

**Figure 1:**
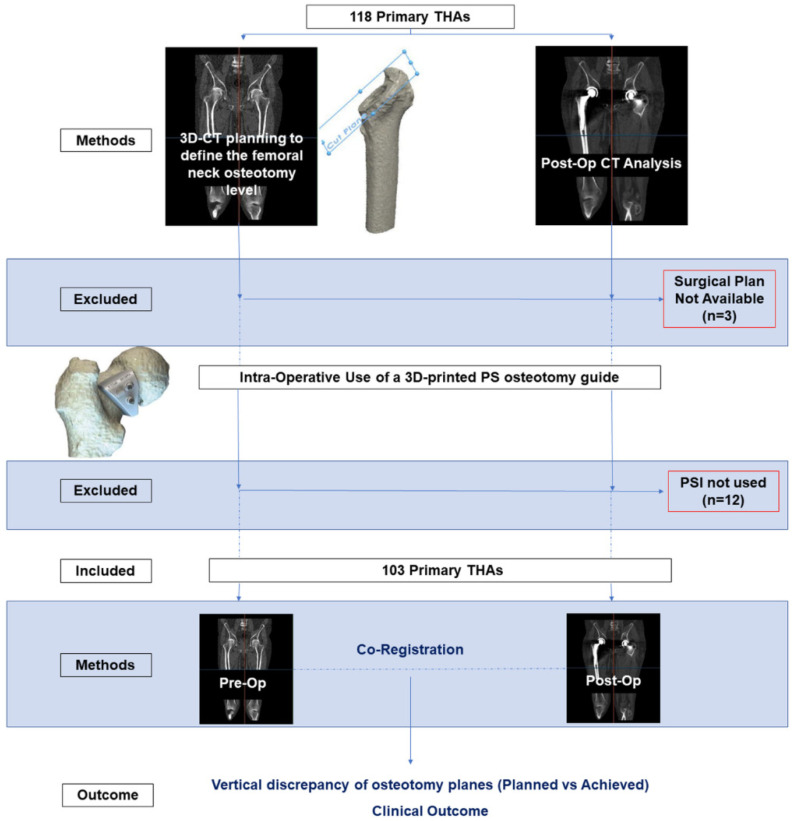
Study design.

Measurements were standardised by means of landmarks selection on the femoral stem translated to the posterior cortex of the proximal femur on the osteotomy plane. The outcome measures were:

1. The relative vertical discrepancy between the achieved and planned osteotomy level.
2. Clinical outcomes including number of dislocations and fractures. (Figure 1 and Table 1)

### 2.2. Pre-Operative Planning and Surgery

**Figure 2:**
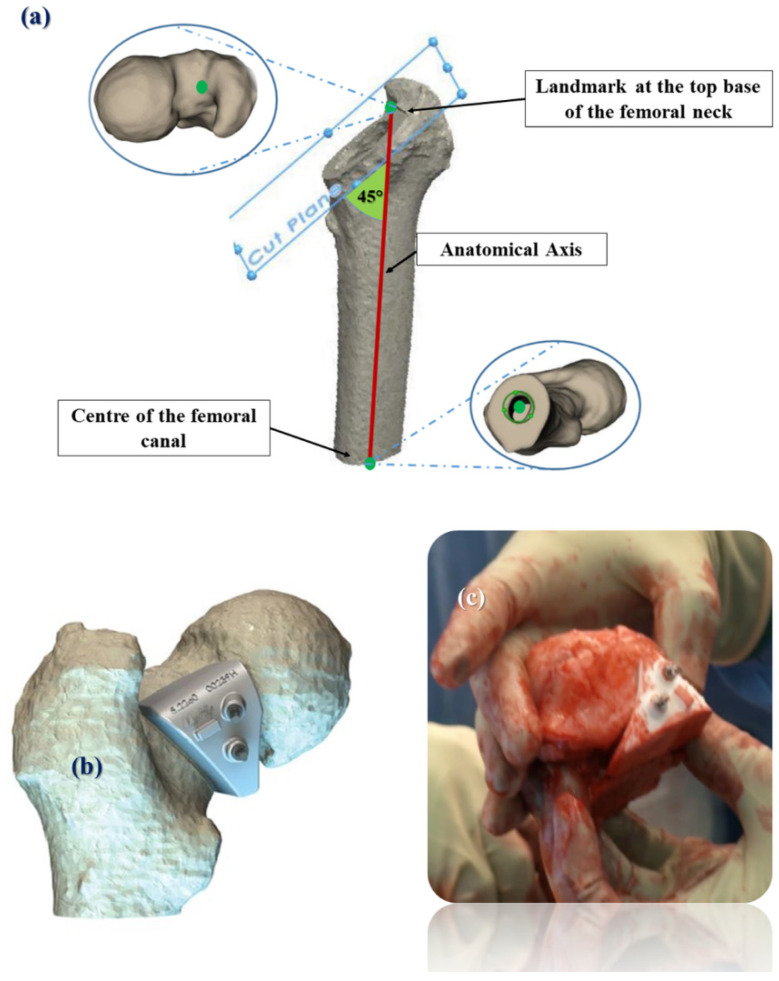
(**a**) Schematic illustration of the surgical plan; (**b**) Illustration of the 3D-printed Patient-Specific (PS) femoral osteotomy guide; (**c**) Two pins secure its position and the surgeon cuts the femoral neck using the saw blade. The femoral head–neck junction is then removed with the guide attached.

Prior to surgery, all patients underwent CT scanning of the hip and the knee joint for surgical planning. The implant manufacturer performed a pre-operative plan using proprietary software (MyHip Planner, Medacta International SA, Castel San Pietro, Switzerland) to establish the acetabular and femoral implant size, position and orientation and define the optimal femoral neck osteotomy level. Selection of the femoral head component aimed to reconstruct the horizontal FO and of the femoral stem to maximize the contact between the bone and the implant. The engineers at Medacta planned the version of the femoral stem relevant to Posterior Condylar Axis (PCA). Cup inclination and anteversion angles were planned at 40° and 20°, respectively. The femoral neck osteotomy plane was defined with reference to the contralateral side, to restore the leg length. The osteotomy angle was planned at 45° relative to the anatomical axis of the proximal femur (Figure 2a). 

All surgeries were performed through a posterior approach by one consultant orthopaedic surgeon. During surgery, two designs of femoral components were used: 1. An uncemented straight-tapered femoral stem in 71 THAs (Quadra-H System; Medacta International SA, Castel San Pietro, Switzerland); 2. A cemented collarless double-tapered femoral stem in 32 THAs (X-acta System; Medacta International SA, Castel San Pietro, Switzerland). 

The design of the PS osteotomy guide used is depicted in Figure 2b. The guide is designed to perfectly fit the patient-specific anatomy (MyHip, Medacta International SA, Castel San Pietro, Switzerland). The time needed for the completion of the surgical plan and manufacturing of the guides is on average of three weeks. The plan is then validated by the surgeon, and the 3D printing manufacturing process follows using Selective Laser Sintering (SLS) of polyamide (PA) 2200, a type of nylon that is a special moulding material developed by EOS (EOS, Munich, Germany). The PS guide is then sterilized for the intra-operative use. 

During surgery, the guide was secured on the femoral neck using two pins. The osteotomy was then performed with the saw blade flush on the plane of the PS guide to remove the femoral head (Figure 2c) [39]. 

### 2.3. Radiological Evaluation

Post-operatively, all patients underwent low dose CT scanning of the hip and the knee joint. Post-acquisition, a normalized metal artefact reduction algorithm (NMAR) was implemented to attenuate the artefacts [40,41]. 3D models of the post-operative femurs were generated using Simpleware ScanIP software (Version 2021.03; Synopsys, Inc., Mountain View, CA, USA).

**Figure 3:**
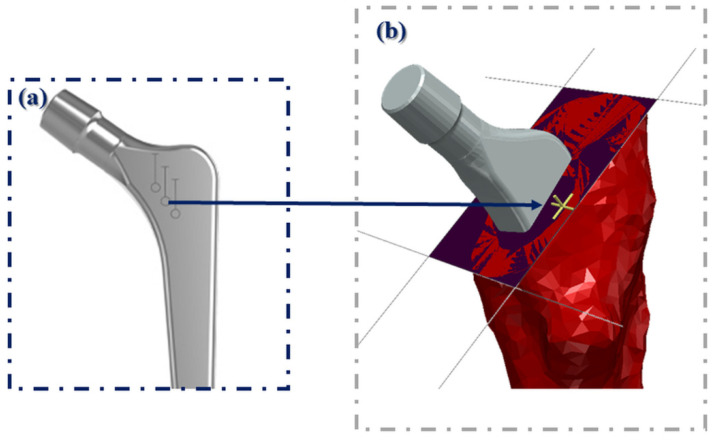
Schematic illustration of the point adopted for evaluation. (**a**) The middle depth marking of the femoral stem, (**b**) was translated to the posterior cortex of the proximal femur on the osteotomy plane.

The osteotomy level was defined as the vertical distance between the Lesser Trochanter (LT) and a systematically defined point at the posterior cortex of the proximal femur; the area that the surgeon uses intra-operatively to evaluate the vertical placement of the femoral stem. This point corresponds to the middle depth marking of the femoral stem, translated to the posterior cortex of the proximal femur on the osteotomy plane (Figure 3).

### 2.4. Pre- and Post-Operative CT Alignment

**Figure 4:**
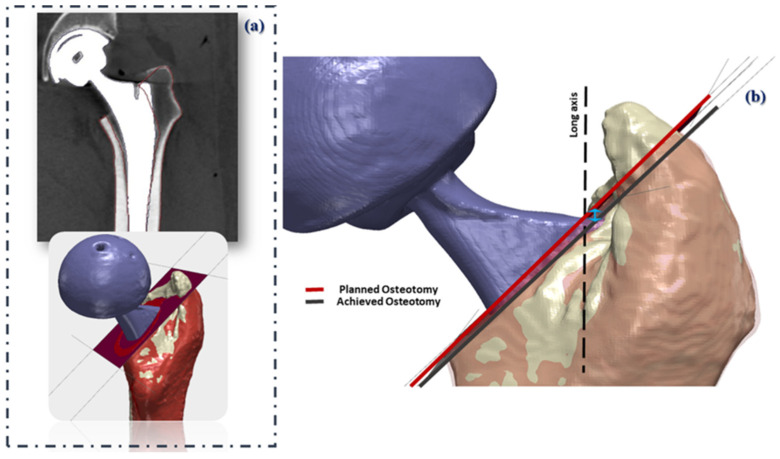
(**a**) The surgical plan in red colour is registered to the post-operative CT scan (model in white colour); (**b**) The relative vertical discrepancy between the achieved and planned osteotomy planes is quantified (light blue arrow).

Alignment of the surgical plan (3D models of the femurs including the planned osteotomy) to the post-operative 3D-CT models was performed through a two-step image registration of the femoral surface (Figure 4a) [40,42]. The relative vertical discrepancy between the achieved and the planned osteotomy levels was subsequently computed (Figure 4b).

### 2.5. Clinical Evaluation

The number of fractures and dislocations was recorded. 

### 2.6. Statistics

Statistical analysis software (SPSS, version 28, Chicago, IL, USA) was used to compute the descriptive statistics for the outcome measures. In order to establish whether the data was normally distributed, the Kolmogorov–Smirnov test (*n* > 50) was utilized. The mean values of the data describing the planned and achieved osteotomy levels were compared using the non-parametric Sign Paired test. A linear regression model was fitted to the data to look for a linear relationship between the planned and achieved osteotomy levels. The coefficient of determination (R^2^) was used to indicate the level of correlation. Statistical outliers were also determined using the Tukey method, abiding by the following conditions:

Outliers < Q1 − (1.5 × IQR) OR > Q3 + (1.5 × IQR)(1)

Q1= 25th percentile

Q3= 75th percentile

IQR = Interquartile Range

## 3. Results

### 3.1. Planned and Achieved Femoral Neck Osteotomy Levels

**Figure 5:**
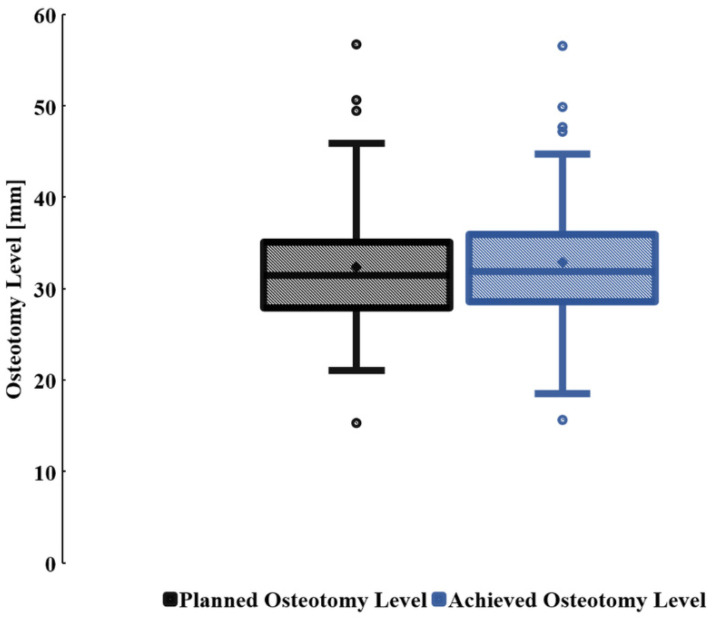
Box plot illustrating the measurements of the planned and achieved osteotomy levels.

The data describing the planned and achieved osteotomy levels did not match the tendency expected for normal distribution (Kolmogorov–Smirnov test, p1 < 0.001; p2 = 0.01). For all patients, the median planned osteotomy level (minimum; IQR; maximum) was 32 mm (minimum = 15 mm; 28 to 35 mm; maximum = 57 mm) and the median achieved osteotomy level (minimum; IQR; maximum) was 32 mm (minimum = 16 mm; 29 to 36 mm; maximum = 57 mm). The mean values of pre- and post-operative measurements did not vary with any statistical significance (*p* = 0.8) (Figure 5). 

### 3.2. Vertical Osteotomy Discrepancy

**Figure 6:**
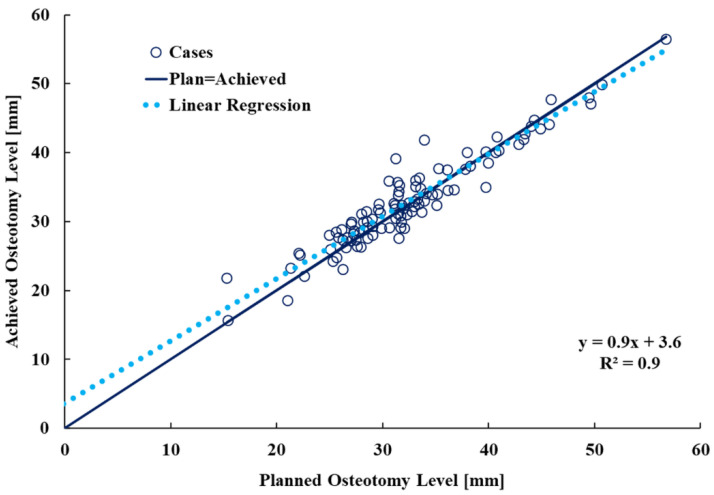
A linear regression analysis plot illustrating achieved osteotomy level as a function of the surgical plan.

**Figure 7:**
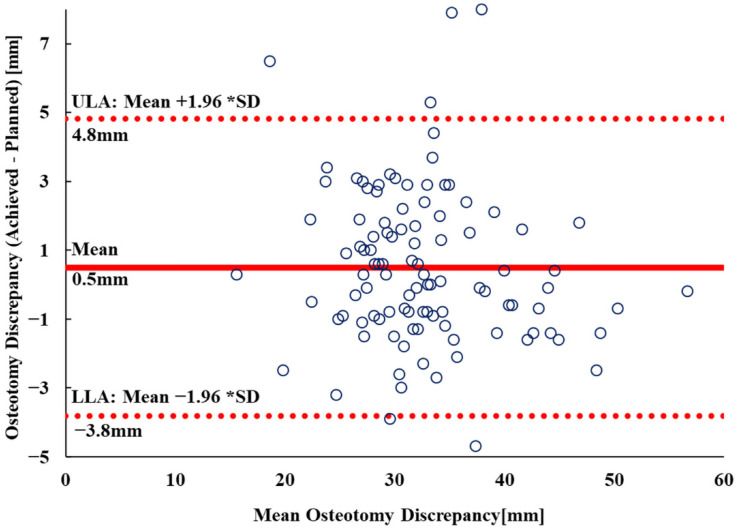
Bland–Altman plot of the comparison between the planned and achieved osteotomy level. The dashed red lines represent the Upper and Lower Limits of Agreements (ULA and LLA). These were 4.8 mm and −3.8 mm, respectively.

The data describing the vertical osteotomy discrepancy and the underlying residuals approached the tendency expected for normal distribution (Kolmogorov–Smirnov test, p1 = 0.2; p2 = 0.1). The median (minimum; IQR; maximum) discrepancy between the achieved and planned osteotomy plane was 0.3 mm (minimum= −5 mm; −1 mm to 2 mm; maximum = 8 mm). A linear regression analysis showed a very strong positive correlation of R^2^ = 0.9 (*p* < 0.001) between the planned and achieved osteotomy level. (Figure 6). A Bland–Altman (BA) plot of the discrepancy between the planned and achieved osteotomy level showed that the 95% limits of agreement (Mean ± 1.96 Standard Deviation-SD) were −3.8 mm and 4.8 mm, respectively. (Figure 7)

**Figure 8:**
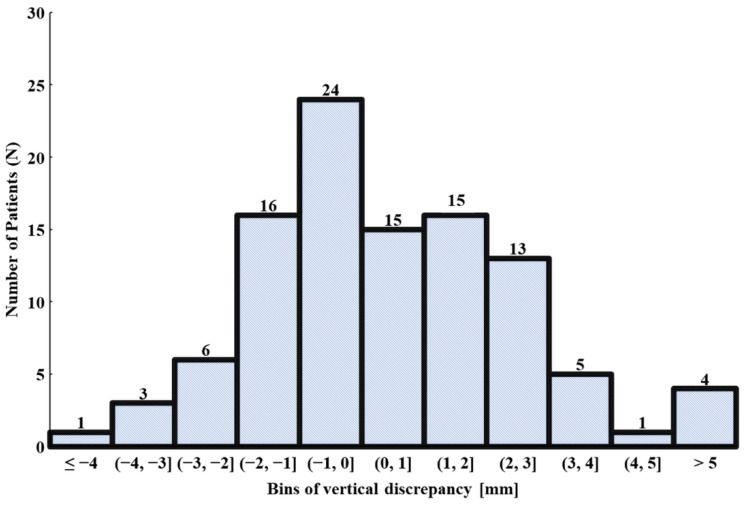
Histogram depicting the distribution of the vertical osteotomy discrepancy in 103 primary THAs.

With regards to the frequency of the osteotomy discrepancy, 96% and 86% of the cases reported an osteotomy discrepancy within 5 mm and 3 mm, respectively. In addition, 68% were within 2 mm, when compared with the surgical plan. In four cases (4%), the osteotomy discrepancy was beyond 5 mm (Figure 8).

### 3.3. Clinical Evaluation

No intra-operative complications such as fracture indicating wrong implant size, have been recorded. There were two dislocations: 1. The first one occurred 5 weeks after the surgery as a result of deep hip flexion; 2. The second one occurred 1 year after the surgery during yoga. Both were treated with a closed reduction procedure with no additional surgery occurred to date. 

## 4. Discussion

Femoral neck osteotomy constitutes the entry point of the femoral stem in THA [4] and that affects its final position and orientation [4,17]. Manual femoral neck osteotomy is conventionally performed by surgeons relying on their experience and finger measurements. This may increase variability and it may be particularly difficult for inexperienced surgeons to deliver an optimal femoral neck resection [18]. PS femoral osteotomy guides have been developed to reduce the uncertainty of femoral neck osteotomy. 

### 4.1. Novelty/Aim/Summarizing Results

This was the first study to assess the accuracy of a PS osteotomy guide in primary THA using 3D-CT analysis in 103 cases. We described a repeatable method to accurately measure the level of the achieved osteotomy plane using imaging computing. 

We found that the 3D-printed guide can be used to achieve a femoral neck osteotomy with a high level of accuracy to the plan. The discrepancy between the achieved and planned osteotomy levels was a median of 0.3 mm and there was a very strong positive correlation between the two measurements (R^2^ = 0.9, *p* < 0.001). 

### 4.2. Comparison with Existing Literature

Limited studies have reported the accuracy of the free-hand osteotomy or femoral guides. Yang et al. (2015) compared the osteotomy accuracy in two groups of patients: 1. A group that received an osteotomy guide; 2. A group that did not receive the guide. They reported a mean osteotomy discrepancy of 0.8 mm when the guide was used and 1.7 mm in the non-guided THA group (*p* < 0.001) [18]. Eggli et al. (1998) have reported a mean osteotomy discrepancy of 4.2 ± 2.8 mm when using conventional templating and no PS guides [43]. In our study, the median (IQR) femoral neck osteotomy discrepancy with the use of a PS guide was 0.3 mm (−1 to 2 mm).

A study addressing the accuracy of a 3D-printed PS femoral osteotomy guide using conventional radiography in 30 patients reported that only 3% of the cases showed an osteotomy discrepancy beyond the clinically accepted threshold of 5 mm [21]. In our study, using a 3D-printed PS guide, 96% of the cases reported a deviation from the surgical plan within 5 mm. We found 4 outliers (4%) beyond 5 mm in a series of 103 cases. 

The existing literature has reported that manual femoral neck osteotomies can affect LLD restoration when compared to guided femoral neck osteotomies. Yang et al. (2015) compared the mean post-operative LLD between a group that received a femoral neck osteotomy guide and one group that did not receive one. The mean LLD in the guided group was 5 mm, while the mean LLD in the non-guided group was 13 mm (*p* < 0.001) [18]. In addition, Sakai et al. (2017) have shown that through the use of PS femoral osteotomy guides, the medial depth of the stem (height of the stem) was more accurate in the PSI group when compared to the control group [44].

### 4.3. Limitations

The presence of outliers clearly highlights that the intraoperative use of a PS osteotomy guide may still induce an error. There are possible factors contributing to this: 3D-printing deviation, malposition of the guide, the presence of soft tissue and misguidance of the oscillating saw, as well as its width, may introduce a significant error. In addition, the PS guide may fit perfectly in more than one location across the femoral head–neck junction inducing a deviation from the surgical plan. Design recommendations may be considered useful to eliminate potential outliers. 

We acknowledge that we performed a non-randomized uncontrolled study. This was a cohort study involving a series of patients that underwent primary THA due to OA, using implants, pre-operative surgical planning software and PSI from a single manufacturer. The accuracy of the PS femoral osteotomy guide was subsequently quantified to evaluate its safety. This impeded the creation of a second group of patients that could be used to compare accuracy. In light of this, we discussed how our findings compare to those reported for surgery without a PS guide.

The prime objective of this study was to assess the accuracy and safety of a commercially available PS femoral osteotomy guide. Five millimetres is the smallest LLD and FO reported difference that has been linked to abnormal gait kinematics [16]. In the present study, 96% of the cases were within this clinically accepted threshold. A prospective multi-centre randomised controlled study is considered imperative to determine if the intra-operative use of a PS femoral osteotomy guide increases accuracy when compared to free-hand osteotomy.

Furthermore, we reported the clinical outcomes, including dislocation and fracture rate as a secondary measurement. We acknowledge that our study did not include a control group or historical data of anticipated frequencies of dislocation or fracture to compare to. However, the aim of the current study was to assess the accuracy of a PS femoral osteotomy guide using CT analysis in a number of hip surgeries. Potential adverse clinical effects were considered essential to be included in the study, despite the absence of a control group or data supporting associations with dislocation or fracture. 

Moreover, 3D-printed PS guides constitute an additional step during a primary THA, which adds a certain cost to the entire process. Sakai et al. (2017) have reported that the manufacturing cost of the PS guide per case is $400 [44], whereas Henckel et al. (2018) have stated that the use of PS guides adds a cost of approximately $371 to each case [20].

We acknowledge further limitations. Although CT may offer greater accuracy compared to conventional radiographs, it is subject to a metal artefact and this may obstruct an accurate definition of the achieved osteotomy plane [45]. However, we implemented a metal artefact reduction algorithm that significantly improved the result. Overall, the processing chain of the implemented method included automated steps with the aim to eliminate the variability of the outcome measures. However, accurate measurements of the outcome values rely on successful registration of the pre- and post-operative CT scans. Based on the results of previous CT studies [40], this step has been reported to be reliable. 

It is true that the angular discrepancy between the planned and achieved osteotomy planes characterises how accurate the PS guide is and may affect the osteotomy level most medially. However, the most clinically relevant outcome measure is the vertical discrepancy of the osteotomy level, which may affect the vertical position of the femoral stem. Even in cases where the vertical discrepancy is moderately variable between the medial and proximal part due to angular discrepancies, the surgeon usually equips a particular area to assess the vertical position of the femoral stem. This area corresponds to the middle depth marking of the femoral stem, translated to the posterior cortex of the proximal femur. In addition, the saw blade is initially positioned at the posterior aspect of the proximal femur, making the selection of that area most suitable to assess the accuracy of the PS guide.

Finally, we evaluated the accuracy of the PS guide using two groups of THAs: 1. One group of patients undergoing uncemented fixation; 2. One group of patients undergoing cemented fixation. Femoral osteotomy using the guide takes place before the implantation of the femoral component and the existence of two different groups following distinct fixation methods was, therefore, assumed not to affect the accuracy of the PS guide.

### 4.4. Future Research

Assessing the accuracy of the PS osteotomy guide is important; major deviations from the plan may contribute to post-operative LLD. However, even an accurate replication of the planned femoral neck osteotomy does not directly imply precise restoration of the leg length and contribution to a satisfactory long-term clinical outcome. A number of variables affect final leg length. Future research should include the evaluation of the relationship between the accuracy of the PS osteotomy guide and the achieved LLD, as well as determining the factors affecting the post-operative LLD.

In addition, PSI femoral osteotomy guides are relatively new in hip arthroplasty, with no long-term clinical studies, raising doubts regarding their contribution to an improved patient clinical response. Currently, there is no evidence that a more accurate femoral cut (either with or without PSI) is associated with an improved clinical outcome or a reduction in post-operative complications. Future research should determine the long-term effect on patient functional outcome over radiation exposure and the associated costs of 3D planning.

## 5. Conclusions

Clinical validation of a PS femoral neck osteotomy guide is important to assess whether the plan is achieved intra-operatively. In this study, we presented a method to measure the achieved femoral neck osteotomy level using 3D-CT analysis. We quantified the discrepancy between the achieved and planned femoral neck osteotomy levels. Our findings suggest that surgeons can use 3D-printed PS guides to achieve the femoral neck osteotomy with a high level of accuracy to the plan. 

## Figures and Tables

**Table 1:** Study Group Characteristics.

	Study Group (*n* = 103 Hips)
Gender (Females) (%)	53 (51)
Age (Years) (Median, Range)	62 (39–89)
Treatment Side (Right) (%)	56 (54)
Follow-Up (Median, Range)	37 (8–66)

## Data Availability

Measurement data presented in this study is available upon reasonable request.
